# Postendodontic Pain after Pulpotomy or Root Canal Treatment in Mature Teeth with Carious Pulp Exposure: A Multicenter Randomized Controlled Trial

**DOI:** 10.1155/2020/5853412

**Published:** 2020-06-30

**Authors:** Mohammad Jafar Eghbal, Ali Haeri, Arash Shahravan, Ali Kazemi, Fariborz Moazami, Mohammad Ali Mozayeni, Eshaghali Saberi, Mohammad Samiei, Mehdi Vatanpour, Alireza Akbarzade Baghban, Mahta Fazlyab, Ardavan Parhizkar, Mahboobe Ahmadi, Nazila Akbarian Rad, Shima Bijari, Delaram Bineshmarvasti, Paria Davoudi, Roya Dehghan, Mandana Dehghani, Habibollah Ebrahimi, Nafiseh Emami, Nafiseh Farajian, Rahim Fereidooni, Gelareh Ghobadi, Mostafa Ghodrati, Atefeh Gohari, Azadeh Hashemi, Mohammadreza Hosseini, Elham Karami, Nasir Kheirabadi, Sepideh Kozegari, Hadi Labaf Ghasemi, Alireza Majidi, Parastu Malekzadeh, Vahid Mehrabi, Mehrnush Mohammadi, Leila Moradi Eslami, Atefeh Noghani, Negin Omatali, Negar Pourhatami, Behnam Rahbani Nobar, Saeid Rahmani, Parviz Shafaq, Sara Soofiabadi, Somaye Teimoori, Farzaneh Vatandoost, Saeed Asgary

**Affiliations:** ^1^Iranian Center for Endodontic Research, Research Institute for Dental Sciences, Shahid Beheshti University of Medical Sciences, Tehran 1983963113, Iran; ^2^Endodontology Research Center, Endodontic Department, Dental School, Kerman University of Medical Sciences, Kerman 7618751689, Iran; ^3^Endodontic Department, Dental School, Shahed University, Tehran 1417755351, Iran; ^4^Endodontic Department, Dental School, Shiraz University of Medical Science, Shiraz 7134814336, Iran; ^5^Endodontic Department, Dental School, Shahid Beheshti University of Medical Sciences, Tehran 1983963113, Iran; ^6^Endodontic Department, Oral and Dental Diseases Research Center, Dental School, Zahedan University of Medical Sciences, Zahedan 9816743463, Iran; ^7^Endodontic Department, Dental School, Tabriz University of Medical Sciences, Tabriz 5166614713, Iran; ^8^Endodontic Department, Dental Branch, Islamic Azad University, Tehran 1946853314, Iran; ^9^Department of Basic Sciences, Proteomics Research Center, School of Rehabilitation, Shahid Beheshti University of Medical Sciences, Tehran 1616913111, Iran

## Abstract

This equivalence, randomized, clinical trial aimed to compare the postoperative pain of root canal therapy (RCT) with pulpotomy with mineral trioxide aggregate (PMTA) or calcium-enriched mixture (PCEM) in permanent mature teeth. In seven academic centers, 550 cariously exposed pulps were included and randomly allocated into PMTA (*n* = 188), PCEM (*n* = 194), or RCT (*n* = 168) arms. Preoperative “Pain Intensity” (PI) on Numerical Rating Scale and postoperative PIs until day 7 were recorded. Patients' demographic and pre-/intra-/postoperative factors/conditions were recorded/analysed. The arms were homogeneous in terms of demographics. The mean preoperative PIs were similar (*P*=0.998), the mean sum PIs recorded during 10 postoperative intervals were comparable (*P*=0.939), and the trend/changes in pain relief were parallel (*P*=0.821) in all study arms. The incidences of preoperative moderate-severe pain in RCT, PMTA, and PCEM arms were 56.5%, 55.7%, and 56.7%, which after 24 hours considerably decreased to 13.1%, 10.6%, and 12.9%, respectively (*P*=0.578). The time span of endodontic procedures was statistically different; RCT = 69.73, PMTA = 35.37, and PCEM = 33.62 minutes (*P* < 0.001). Patients with greater preoperative pain, symptomatic apical periodontitis, or presence of PDL widening suffered more pain (*P*=0.002, 0.035, and 0.023, resp.); however, other pre-/intra-/postoperative factors/conditions were comparable. Pulpotomy with MTA/CEM and RCT demonstrate comparable and effective postoperative pain relief.

## 1. Introduction

Dental caries, as a chronic infectious disease, is a global health concern. The untreated caries is the most prevalent condition (global prevalence = 34.1%) evaluated for the entire “Global Burden of Disease”, as it remains the main cause for loss of all teeth [1]. Endodontic diseases, as the sequelae of untreated caries, affect oral health-related quality of life with moderate severity, mainly through physical pain of pulpitis [2]. A systematic review showed that the mean prevalence of preoperative endodontic-associated pain was as high as 81% [3]. Therefore, endodontic pain can considerably affect the quality of life. In addition, endodontic pain was the most prevalent self-reported reason for tooth extraction [4].

Pain research has gradually expanded its prominence in all medical- and dental-related sciences. In endodontology, pain control in intra- and postoperative phases is a key aspect of clinical practice. Despite novel advancements in endodontology, up to 58% of patients have experienced postendodontic pain [5]. The postoperative endodontic pain is a multifactorial phenomenon. Previous researches have tried to correlate it with predictive factors such as the patient- and tooth-related determinants as well as pre- and intraoperative conditions [3, 6, 7]. However, results have often been uncertain, unpredictable, or lacking obvious “cause and effect” relationship [2].

The single available Cochrane review, appraising pulp management in carious permanent mature teeth with inflamed pulp, was not able to reach a conclusion and thus stated that further well-designed randomized controlled trials would be compulsory [8]. Recently, endodontology has focused on biologically based restorative solutions [9] for the management of extremely deep caries, so that it could preserve pulpal vitality/health and the integrity of tooth structure with minimally invasive approaches for instance vital pulp therapy (VPT) [10]. According to the “European Society of Endodontology (ESE) position statement” in extremely deep caries lesion, the caries entering the entire thickness of the dentine and the pulp exposure is unavoidable during operative treatment [11]. In carious pulp exposures, if inflammation/infection is effectively controlled, VPT, employing endodontic biomaterials, can provide a biological seal against bacterial microleakage and promote the noninflamed pulp to create dentine-like hard tissue [12, 13]. Unlike RCT, VPTs are not complex, lengthy, and costly; however, like RCT, they are successful, acceptable, accessible, and available [14]. Given the growing evidence supporting the clinical effectiveness of VPT, RCT is no longer the treatment of choice for the management of carious pulp exposures, although it may still be the routine practice. Furthermore, the advantages of VPT over RCT are not only the preservation of tooth structure but also the maintenance of pulp's mechanoreceptors, regeneration capacity, and proprioceptive feedback [15].

An ideal endodontic biomaterial for VPT must be biocompatible and antibacterial, able to provide a biological and bacterial-tight seal, and promote the regeneration of dentine-pulp complex [16]. Mineral trioxide aggregate (MTA) and calcium-enriched mixture (CEM) cement are two bioactive endodontic cements (BECs) with different chemical compositions [17] which are mainly comprised of calcium and silicate elements. MTA and CEM cement demonstrated similar antibacterial effect [18] and comparable sealing ability [19]. In terms of bioactivity and biocompatibility, these BECs are able to induce hard tissue formation, i.e., osteogenesis [20], dentinogenesis [21], and cementogenesis [22]. Based on such favorable properties, they are employed for VPTs with promising results [23].

A large noninferiority open-labelled clinical trial revealed that when general dentists treated cases with irreversible pulpitis, the pain-reducing effect of CEM pulpotomy (PCEM) was considerably more than that of RCT using hand instrumentation techniques [24]. Moreover, a recent randomized controlled trial reported that, in managing symptomatic permanent teeth with extremely deep caries lesions, in comparison with RCT, MTA pulpotomy (PMTA) effectively reduced postoperative endodontic pain [25]. A quadruple blinded randomized controlled trial also showed the comparable pain relief effect of PMTA and PCEM [26]. Another randomized clinical trial showed that the levels and incidence of postoperative endodontic pain were lesser in rotary instrumentation groups compared with manual preparation groups [27]. Hence, the aim of this randomized controlled trial was to compare the pain reduction of CEM or MTA pulpotomy with RCT using rotary instrumentation techniques; when postgraduate endodontic students managed extremely deep caries of mature permanent molars with/without clinical signs of irreversible pulpitis and apical periodontitis. The null hypothesis was, in terms of postoperative endodontic pain relief, PMTA/PCEM would be equivalent to RCT.

## 2. Materials and Methods

The Iranian Ministry of Health and Medical Education (IMHME) commissioned this project. The trial has been managed by the Iranian Center for Endodontic Research (ICER) and Research Institute for Dental Sciences (RIDS) of Shahid Beheshti University of Medical Sciences (SBMU), Tehran, Iran. The project was registered in the Iranian Registry of Clinical Trials (Registration Number IRCT20151226025695N3 and Registration Time 06 Feb., 2019) and approved by the Ethics Committee of Research Institute for Dental Sciences (IR.SBMU.RIDS.REC.1395.320) and Shahid Beheshti University of Medical Sciences (IR.SBMU.RETECH.REC.1397.1187). All experimental protocols were approved by RIDS (29-1395/09/23) and SBMU (10466-1396/04/26).

The trial was conducted in accordance with the ethical principles of the “Declaration of Helsinki” and “General Ethics Guideline” in Medical Sciences Research (which has a human subject) in Iran.

### 2.1. Resources

The research grant was funded by the Deputy Minister of Research, IMHME. The funder had no role in the design/conduct of the trial and analysis of the data.

### 2.2. Design of the Study

The project was a multicenter, randomized, open-label, clinical trial.

### 2.3. Hypothesis

For this part, we hypothesised that pulpotomy with endodontic biomaterials (MTA or CEM cement) would be considered as an equivalent to root canal therapy, employing rotary instrumentation, in terms of postoperative pain relief.

### 2.4. Objective

In this phase of the study, the primary outcome was to assess whether full pulpotomies with MTA or CEM cement decreased postoperative pain in permanent mature teeth with cariously exposed pulp compared with RCT.

Secondary outcomes were to assess probable associations between the following determinants and pain relief in cariously exposed mature teeth:

  Patients' demographics (i.e., age, gender, level of education, and marital status)
  Preoperative factors (i.e., baseline pain intensity/dental characteristics and pulp/periapical response to diagnostic tests/diagnosis)
  Intraoperative factors (i.e., type of pulp exposure, type of pulpal hemorrhage with/without chlorhexidine/NaOCl, number of treatment sessions, and time-length of endodontic/restorative procedures)
  Postoperative factors (pulp/periapical response to diagnostic tests and type/number of prescribed analgesics)

It should be noted that the main endpoint of the second phase of trial would be the two-year radiographic outcomes.

### 2.5. Criteria for Patient Selection

Volunteers were recruited from the pool of referred patients to seven postgraduate “Departments of Endodontics” in Iran. All patients had to adhere to inclusion and exclusion criteria.

### 2.6. Inclusion Criteria

Inclusion criteria were the following:

  Vital molar teeth (confirmed with cold spray or electric pulp tester before treatment and visual inspection of pulpal hemorrhage after access cavity preparation)
  A history of pain, indicating irreversible pulpitis (i.e., spontaneous localized/generalized pain, pain stimulated by hot/cold fluids that lasts after elimination of the stimulus and is reproducible with cold sensibility testing)
  Pulp exposure during caries removal (when no clinical sign of irreversible pulpitis was present)
  Patients in the range of 12 to 65 years of age
  Patients who accepted to be available for recalls
  Patients who approved and signed the written informed consent.

### 2.7. Exclusion Criteria

Exclusion criteria were the following:

  Nonvital/partially necrotic teeth (confirmed with primary examinations or diagnosed after access cavity preparation)
  Teeth with continuous bleeding (after placement of a cotton pellet soaked in sodium hypochlorite for a maximum of 10 minutes)
  Teeth that were not appropriate candidates for class I or II restorations and required complicated build-ups, crown lengthening, or prosthetic crowns
  Teeth with localized/generalized periodontal diseases (i.e., depth of probing ?3 mm)
  Internal/external root resorption or detectable pulp chamber/canal calcification
  Teeth with history of trauma
  Immature teeth with open apices
  Patients with systemic conditions that disturbed tissue healing process (i.e., diabetes, cancer, AIDS, intake of corticosteroids, and so forth)
  Pregnant/nursing women
  Physically/mentally disabled patients
  Patients with poor oral hygiene
  Immigrants, who were likely to leave the country during the subsequent two years
  Patients younger than 12 years of age for the first molar treatment, 17 years of age for second molar treatment, and older than 65 years of age.

### 2.8. Informed Consent

Informed consent was obtained from all participants and from parents/legal guardians for patients under the age of 18 years.

### 2.9. Randomization

Patients were allocated in the three study arms with simple randomisation. Randomisation was performed online (http://www.mcrct.ir) immediately before the commencement of the treatment. After recording the patient's demographics, eligible patients, using a computer-generated system and under the supervision of administrative staff, were automatically randomised into different study arms including PMTA, PCEM, and RCT. All the allocated patients received a unique patient identification code before starting the treatment. In the event of withdrawal from the study, the randomisation code was not reused. Patient blinding was not implemented; however, the assessors and analysers were blinded.

### 2.10. Treatments

#### 2.10.1. PMTA/PCEM

Each patient used a 0.2% chlorhexidine mouth rinse (Iran Najo Pharmaceutical Co., Tehran, Iran). Then, the teeth were anaesthetized with 2% lidocaine and 1 : 80000 epinephrine (Darou Pakhsh, Tehran, Iran). Upper and lower teeth were anaesthetized using infiltration and inferior alveolar nerve block techniques, respectively. Afterwards, cavity preparation and soft dentine removal were carried out using appropriate sterile high-speed diamond and low-speed large round burs, respectively. Subsequently, the tooth was isolated using a rubber dam and disinfected with 0.2% chlorhexidine.

Complete pulpotomy was performed by means of a new sterile long cylindrical round-ended diamond bur (Tiz Kavan, Tehran, Iran) in a high-speed handpiece with copious water irrigation. To achieve hemostasis, a cotton pellet moistened with 0.2% chlorhexidine was placed into the pulp chamber for 5 min; and, if needed, it was replaced with a cotton pellet moistened with NaOCl 5.25% for 30 sec, or in cases of excessive hemorrhage, for 10 minutes. The pulp-covering agent (MTA, ProRoot, Dentsply, OK, USA, or CEM cement, Bionique Dent, Tehran, Iran) was prepared according to the manufacturer's instructions and then inserted into the pulp chamber. Using a dry sterile cotton pellet, the biomaterial was gently adapted on the blood clot-free pulpal wound and dentinal walls with a thickness of approximately 2–3 mm. Within the same session (after the placement of a moistened cotton pellet for 5 min) or further appointments, the cavity was permanently filled with Glass Ionomer (ChemFil, Dentsply, Konstanz, Germany) and light-cured resin-bonded dental composite (Filtek flowable/Z250/Z350, 3M, ESPE, USA), employing open/close sandwich technique.

#### 2.10.2. RCT

Similar to two other arms, teeth were anaesthetized and isolated, and pulpotomy was conducted. Then, working lengths were determined using Root ZX apex locator (J. Morita, Irvine, CA) and established 1 mm short of the apex employing parallel technique (X-ray Holder, Kerr Corporation, Orange, CA, USA). A digital radiograph confirmed the working lengths.

Root canal preparation was carried out using BioRaCe instruments (FKG Dentaire, La Chaux-de-Fonds, Switzerland). The endodontic motor (Endo-Mate TC, NSK, Nakanishi Inc., Tokyo, Japan) was adjusted at 600 rpm and 1.5 Ncm. Rotary files, with recommended sequences/motions, were employed in a crown-down manner according to the manufacturer's instructions. Root canals were irrigated with copious amounts of 5.25% NaOCl. Canal patency was confirmed with a #10 K-file during the procedures. Master apical file usually ranged to BR4 (35/0.04) or BR5 (40/0.04) for straight canals or BR4C (35/0.02) for curved canals. After radiographic confirmation of master cone, the canals were dried with sterile paper points (Ariadent, Tehran, Iran). Using cold lateral condensation technique, root canals were filled/sealed with multiple gutta-percha cones (Ariadent, Tehran, Iran) and AH-26 resin-based sealer (Dentsply, Tulsa Dental, Tulsa, OK, USA). Within the same/further sessions, the cavity was permanently filled employing sandwich technique.

### 2.11. Sample Size Calculation

Sample size was calculated based on the two-year outcomes in previous studies [28, 29]; the two-year radiographic success rates of PMTA, PCEM, and RCT were approximately 94%, 86%, and 80%, respectively. Using PASS 11 software, considering the effect size = 0.168, *α* = 0.05, and *β* = 0.1 (power = 90%), the estimated sample size was 449.

### 2.12. Role of Participating Centers

Postgraduate departments of endodontics in seven universities of medical sciences, located in five states of Iran, collaborated in this multigroup collaboration. Thirty-four postgraduate students (PGS) with similar experience/skills participated in the study. The selected PGSs attended a training workshop at ICER, which included the demonstration of the study protocol, hands-on training in RCT, and protocols on full pulpotomy treatment. Then, PGSs took/passed the final test and were considered qualified for the trial. The primary task of each PGS was to recruit nine patients and fulfill including/excluding criteria in each study arm. One endodontist from the academic staff of each department agreed to supervise all processes and activities, i.e., accordance of including/excluding criteria, standardisation of treatment, assessment of the outcomes, and recording of the data. The included centers were Azad Tehran (PGS = 6), Kerman (PGS = 5), Shahed (PGS = 2), Shahid Beheshti (PGS = 6), Shiraz (PGS = 6), Tabriz (PGS = 6), and Zahedan (PGS = 3) schools of dentistry.

### 2.13. Data Recording

Similar to previous studies [24], using a published pain “Numerical Rating Scale” (NRS) with ratings from 0 to 9 within four grades (pain-free, mild, moderate, and severe), each patient recorded the pre- and postendodontic pain intensities at 11 time intervals. The PGS, with no assessment/alternation of the data, entered them directly into the trial database http://www.mcrct.ir. The database was inaccessible if the related data for each patient did not timely enter. At the end of study, coded data without the names of patients and treatment groups were extracted and analysed.

### 2.14. Statistical Analyses

The analysis of data was performed using SPSS software (SPSS 21, SPSS Inc., Chicago, IL, USA). The comparisons were considered significant if *P* < 0.05.

A comparison of baseline demographic data was performed with one-way analysis of variance test (ANOVA) for age and the Pearson chi-squared test (*χ*^2^) for age categories, sex, marital status, years of schooling, and distribution of treated teeth.

Data for preoperative conditions including preoperative PIs, dental characteristics, preoperative pulp sensibility tests, and presence of apical periodontitis [PDL widening (periapical status was assessed using the periapical index [30]) or positive response to percussion test] were tested using ANOVA, *χ*^2^, and Fisher Exact Test (FET).

Data for intraoperative factors, i.e., type of pulp exposure, type of bleeding, irrigants usage for hemostasis, length of treatment, and number of treatment sessions, were analysed using Kruskal Wallis, ANOVA, *χ*^2^, and FET.

Data for postoperative factors, including postoperative pulp sensibility tests and analgesic intake, were tested using independent *t*-test, ANOVA, *χ*^2^, and FET.

The postoperative pain intensity, trend, distribution of severity, and survival in the study arms were compared using one-way/repeated measure ANOVA, ANCOVA, Kruskal Wallis, and Kaplan-Meier (log-rank) test.

## 3. Results

**Figure 1:**
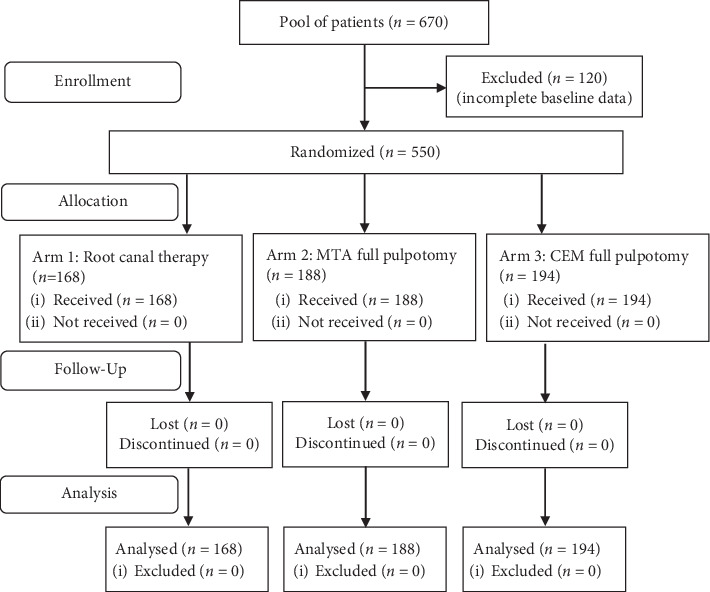
CONSORT flow diagram of participants through each stage of randomized clinical trial.

Five-hundred fifty participants that met the inclusion criteria agreed to be randomised in the study arms: 188 (34%) in PMTA, 194 (35%) in PCEM, and 168 (31%) in RCT (Figure 1). They were recruited from seven academic endodontic departments in seven dental schools, located in five states of Iran. Patients' recruitment started from March 2017 and ended in March 2018. All participants finalised the follow-up for 7 days. Participant demographics, i.e., age, age categories, gender, marital status, and level of educational, were normally distributed and were homogeneous (*P* > 0.05) (Table 1).

Baseline characteristics and preoperative conditions, i.e., preoperative PI, distribution of treated teeth and their characteristics (presence or absence of occlusal contact/occlusal attrition/coronal restoration), the results of clinical/radiographic examinations (sensibility testing), and final clinical diagnosis, in all experimental arms, were similar (*P* > 0.05) (Table 2).

Except number of treatment visits (*P* < 0.001) and the treatment length of endodontic procedures (*P* < 0.001), all intraoperative conditions, i.e., type of pulpal exposure, nature of bleeding, achieving hemostasis, treatment length of restorative procedures, and number of treatment sessions, were similar in the three study arms (*P* > 0.05) (Table 3).

Postoperative conditions, i.e., response to sensibility testing (only in pulpotomy arms), percussion tests, and analgesic intake, were comparable in all study arms (*P* > 0.05) (Table 4).

**Figure 2:**
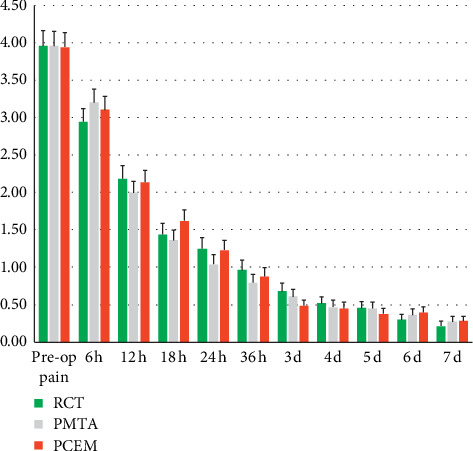
Pain intensities during the study in the RCT, PMTA, and PCEM arms.

Repeated measure ANOVA revealed that whilst PI changes, during post-operative 7 days, were statistically significant (*P* < 0.001), the trend of PI scores amongst three study arms was similar during the study (interaction effect, *P*=0.821). There was no difference between arms for the quantity of pain relief (*P*=0.947) (Figure 2). In addition, the scores of preoperative PI were correlated with experienced postoperative sum PIs (*P*=0.002).

**Figure 3:**
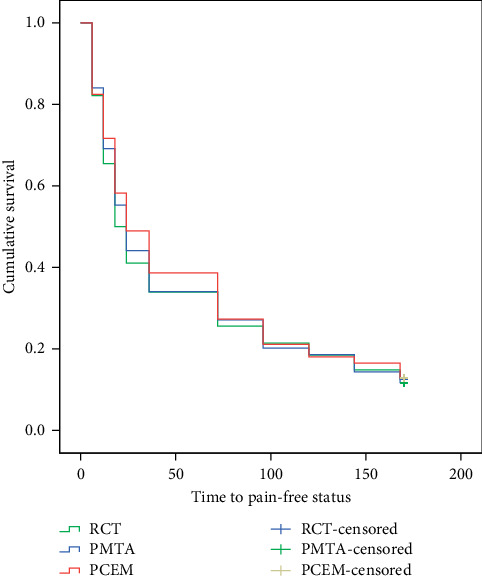
Time to pain-free status in the three study arms.

In terms of grouping pain severities (i.e., pain-free status, mild, moderate, and severe pain) in different postoperative time intervals, Kruskal–Wallis test revealed that there were no significant differences between the study arms (*P*=0.496–0.942). In addition, the distribution of pain severities was comparable between the arms (*P*=0.056–0.993) (Table 5). The mean and median time to pain-free status in the three study arms were from 54 to 58 h and 18 to 24 h, respectively, with no significant difference (Table 6, Figure 3).

**Figure 4:**
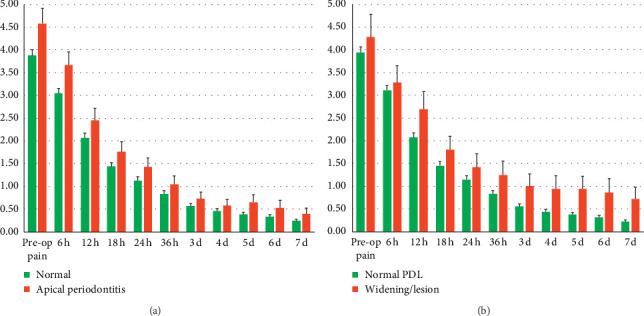
Pain intensity changes in presence or absence of (a) apical periodontitis or (b) PDL widening/lesion.

Repeated-measures ANOVA with postoperative PIs during the study as within-subjects factors, sex, marital status, age groups, and level of education as between-subjects factors showed that there were no significant effects (*P*=0.626, 0.197, 0.834, and 0.386, resp.). When dental characteristics were set as between-subjects factors, the test revealed that there were no statistical differences for the presence or absence of occlusal contact, occlusal attrition, and coronal restorations (*P*=0.702, 0.393, and 0.059, resp.). When presence or absence of clinical apical periodontitis (positive response to percussion test), detection of PDL widening/lesion, and diagnosis of irreversible pulpitis were set as between-subjects factor, the test revealed that cases with apical periodontitis or PDL widening/lesion significantly suffered more pain (*P*=0.035 and 0.023, resp.) (Figure 4). However, there was no such pattern for irreversible pulpitis cases (*P*=0.782). In addition, severity of hemorrhage after pulp exposure, as well as hemostasis achievement after application of CHX, had no influence on postoperative PIs (*P*=0.452 and 0.438, resp.). Finally, other factors/conditions had no significant effect on postoperative PI in the study arms (*P* > 0.05).

## 4. Discussion

Pulpal pain, as a major component of oral health-related quality of life, is often the stimulus for patients to seek endodontic care [31]. In addition, postendodontic pain relief is an important concern when evaluating endodontic treatment alternatives, i.e., VPTs [24]. The present randomised clinical trial studied the pain relief effect of PMTA/PCEM as an alternative treatment modality in comparison with RCT (with rotary instrumentation) as a standard protocol, in the management of cariously exposed dental pulp in symptomatic/asymptomatic mature molars. The obtained results revealed that, as well as RCT, postendodontic pain relief of new biological treatments, i.e., PMTA/PCEM, was highly effective.

Bacterial invasion is often the cause for pulpal inflammation, which is considered as the cause of toothache. Therefore, the pulpal pain should be reduced if (i) the irritating factors are eliminated, (ii) the inflamed pulp is removed, and (iii) the surgical wound is protected by antibacterial BECs [32]. Previous research showed that coronal pulpotomy, using MTA or CEM cement, effectively controlled the preoperative pain in cases with irreversible pulpitis [26].

In all studied arms, mean preoperative PI scores were comparable (approximately 4 on the scale of 0–9 in NRS), which were similarly decreased to approximately 1 and 0.5, one and seven days after treatments, respectively. In addition, the median time to pain-free status for all patients was 18–24 h. These results indicated that pulpotomy with BECs could be considered as effective as RCT in postendodontic pain relief; and, thus, the null hypothesis was accepted. These findings are supported with those of a systematic review which showed that moderate pretreatment root canal-associated pain severity dropped significantly within 1 day after treatment and continued to be lower to minimal levels in 7 days [3].

In the present trial, patients with symptomatic apical periodontitis or detected PDL widening/lesion experienced more postoperative pain. In addition, the preoperative PIs were significantly correlated with postoperative PIs. Amongst several predictive factors for postendodontic pain, including patients' age/gender/level of education/marital status, tooth type/location, pulp/periapical status, types of intracanal dressings, systemic analgesic/corticosteroid/antibiotics intake, type of anaesthetics, number of treatment sessions, and different treatment procedures, the presence of preoperative pain is a major predictor of postoperative pain after endodontic treatments [7, 33, 34], which supports our obtained results.

Whilst mean baseline PI scores in this trial were classified as mild, approximately 20–22% of recruited cases reported severe preoperative pain. 24 hours after RCT (using rotary files) or VPTs, such preoperative pain dramatically decreased to roughly 1–3% of all patients, with no statistically significant difference between the arms. A previous noninferiority clinical trial on irreversible pulpitis demonstrated the effect of PCEM and one-visit RCT on pain reduction; however, when general dentists treated the teeth, pulpotomy arm significantly had faster/more pain relief than RCT using hand instrumentation techniques [24]. Researchers reported that, compared with coronal pulpotomy, emergency pulpectomy resulted in higher incidence of postoperative pain [35]. A probable source for postendodontic pain is periapical tissue contamination and irritation, initiated by endodontic instrumentation [36]; when dentists were trained to replace manual techniques with nickel-titanium rotary instrumentation, the quality of RCT increased [37]. In addition, with shifting the provider from general dentists to endodontists, treatment outcomes in endodontically treated teeth improved [38]. Thus, clinical expertise as well as the type of the employed techniques may explain such differences.

In this trial, in addition to cases with carious pulp exposure, all cases with clinical signs of irreversible pulpitis were included. Based on the results of the cold test, 23.8% of enrolled patients were diagnosed with symptomatic irreversible pulpitis. However, postoperative pain relief between patients with or without initial diagnosis of symptomatic irreversible pulpitis was similar. It was assumed that irreversible pulpitis is a clinical term, which indicates that the painful vital inflamed pulp is incapable of healing and needs to be treated with RCT [39]. Currently, new understanding of biology regarding dentine-pulp complex clearly shows that human dental pulp, with irreversible pulpitis, has putative stem cells [40]. In this context, presence of inflammation is not an undesirable phenomenon as it is a prerequisite for pulp healing/regeneration like all the other body connective tissues [41]. Numerous clinical trials have shown that teeth, clinically diagnosed with irreversible pulpitis, could be successfully managed by VPTs [29, 42–45]. Currently, the growing body of evidence has established that clinical sign/symptoms of irreversible pulpitis are not a contraindication for VPTs [44, 46, 47]. These attitudes indicate an outdated classification for dental pulp diseases [10, 31, 48]. Furthermore, VPTs can significantly reduce the preoperative pain of irreversible pulpitis cases [24, 26]. Based on these new findings and high-level evidence within this field, it seems that the classification of dental pulp diseases, as well as indication of treatment options, needs a major reconsideration.

At the baseline, although approximately 70–73% of cases, in all study arms, responded to the cold test, almost all cases responded to EPT (98.8–100%). One week after full coronal pulpotomy, these responses to the cold test and EPT decreased to approximately 22–26% and 48–52%, respectively. Amputating the coronal dental pulp with rich subodontoblastic plexus of low-threshold myelinated A-delta sensory afferent fibers and replacing it with BEC, which is expected as an insulator, and maintaining the radicular pulp with high-threshold C sensory nerve endings, which are located far from coronal tooth surfaces [49], may explain the lesser pulp response to each sensibility testing. In addition, histological studies revealed that, two months after coronal pulpotomy with MTA or CEM cement, hard tissue formation took place at canal orifices [12, 50]. This could probably compromise the responses to sensibility testing with the passage of time and, if needed, complex the further endodontic interventions. It should be noted that diagnostic accuracy of these routine clinical sensibility tests might not be valid for the determination of dental pulp condition [31]. In order to obtain accurate diagnosis of dental pulp status, the sensibility tests should be replaced with pulp vitality tests, i.e., pulse oximetry and laser Doppler flowmetry [51]. These diagnostic technologies mainly focus on pulpal blood supply, not the nerve supply.

Inability of paying for dental services while suffering from endodontic pain [52] and fear of root canal treatments are two major concerns for patients [2]. Obtained results showed that whilst the duration for restorative procedures was comparable in all study arms, duration of pulpotomy was much lesser than RCT (33–35 vs. 69 minutes). In addition, although almost all patients in pulpotomy arms received both endodontic and restorative treatments in one session, 29% of patients, who underwent RCT, were managed in two sessions. In comparison with pulpectomy/RCT, simplicity of VPTs minimised the fear and time needed for the treatment and caused minimisation in treatment cost [28, 53]. Even though the treatment effect of RCT and pulpotomy produced equivalent postoperative pain reduction, new biotechnology using BECs might be desirable [54], especially for patients and more specifically for people with low socioeconomic status. If the long-term outcomes of this trial reconfirm the present short-term results, the simple and cost-effective VPT biotechnology [55], employing endodontic biomaterials, i.e., MTA or CEM cement, might become more popular.

In this trial, regardless of nature of bleeding, PGSs applied 0.2% chlorhexidine solution on all amputated pulps for 5 minutes to achieve hemostasis. However, in approximately 44–56% of cases, bleeding lasted longer than 5 minutes, which were effectively controlled with the application of full strength NaOCl for 30 seconds. In clinical practice, dentists and specifically pedodontists usually shift vital pulp therapy to pulpectomy or even tooth extraction if excessive hemorrhage is evident after pulp amputation or even at the site of pulp exposure [56]. Basically, in response to microbial irritation, blood-vessel vasodilation and subsequent hyperemia, as the signs of inflammation, occur to help pulpal cells/tissue with better blood supply [57]. Therefore, when effective elimination of etiological factors along with further protection of remaining pulp with antibacterial/biological sealant takes place, such blood overflow may be beneficial in healing process.

Toothache, as the chief complain of patients, can be definitely treated through extraction or RCT [58]. The latter rapidly causes a dramatic reduction in preoperative endodontic pain [3]. However, endodontic treatment outcome studies have not primarily focused on pain relief as the most exasperating factor for the patient but rather concentrated on the radiographic success, which have two pitfalls. First, many apical periodontitis cases without visible radiographic signs (in the two-dimensional dental radiographs) cause underestimation of actual failure rate of endodontic treatments [59]. Second, long-term radiographic evaluation fails to address the issues of primary concern to patients, i.e., pain.

Participants, without being excluded from the study, were permitted to take effective analgesics if needed postoperatively. The number of people who used analgesics, mean number of taken tablets/persons, and selected drugs were comparable in the study arms. The commonly taken analgesics were gelofen, ibuprofen, and acetaminophen, respectively. A recent systematic review revealed that administration of nonsteroidal anti-inflammatory drugs (NSAIDs) and/or paracetamol was effective in the management of postendodontic pain [60].

In this multicenter randomized controlled trial, the calculated sample size with 90% power was equal to be 449; yet 550 patients were enrolled. As the sample size increases, the minimum point of the power function moves toward one [61]. An appropriate large sample size provides a high power for statistically significant evidence for the efficacy. In addition, based on the nature of our trial, each patient was in his/her own control and preoperative pain and postoperative PIs measures (as effect) were collected with the same scale (NRS). Moreover, and to reduce bias, randomisation was carried out immediately before initiation of treatment procedure using an online computer-generated system. Due to appropriate randomisation process and allocation concealment, the patients' demographics, tooth type/location, and pulp/periapical diagnosis were similar and homogeneous in all experimental arms. Furthermore, the effect of variables that may have had probable association with the postendodontic pain relief was analysed. The above-mentioned items enhanced statistical conclusion validity of the trial.

Although the trial was a multicenter study, it ran in a country with single race (white/Caucasian). It has been reported that chronic pain could be associated with race [62]. Therefore, generalization of obtained results to other races probably needs further investigations.

Our study had two limitations. First, the trial had open-labelled design; and, due to the differences between treatment methods and materials, conducting a blinded study was not possible. Second, we did not assess the socioeconomic status of participants, whilst this factor might be associated with dental pain [63]. However, due to large sample size and appropriate randomisation in our trial, it is expected that this variable, such as other patients' demographics, may be evenly distributed in the three study arms.

A reported adverse side effect, after pulpotomy using calcium-silicate materials, was the potential of tooth discolouration [64]. Grey discolouration of the treated tooth was noticeable after seventeen months [65]. Researchers reported that another unpredictable reaction after coronal pulpotomy was deposition of hard tissue inside the root canals, i.e., pulp obliteration in 30% of treated cases during 62 months [66]. However, our trial in this phase was graded as short-term one and therefore such potential discolouration and pulp obliteration were not assessable.

## 5. Conclusion

In the management of symptomatic/asymptomatic mature permanent molar teeth with cariously exposed pulp, PMTA/PCEM and RCT demonstrated effective and comparable postoperative reduction of pain.

## Figures and Tables

**Table 1:** Comparison of patient demographics in the three study arms.

Demographics	RCT (*n* = 168)	PMTA (*n* = 188)	PCEM (*n* = 194)	Test	*P* value
Age (mean years ± SE)	29.59 ± 0.83	28.89 ± 0.77	29.77 ± 0.75	ANOVA	0.70

Age category, *N* (%)					
10 ≤ age ≤ 20	33 (19.6)	40 (21.3)	33 (17.0)	*χ* ^2^	0.894
20 < age ≤ 30	53 (31.5)	66 (35.1)	72 (37.1)		
30 < age ≤ 40	54 (32.1)	52 (27.7)	57 (29.4)		
40 < age ≤ 50	20 (11.9)	25 (13.3)	25 (12.9)		
50 < age ≤ 61	8 (4.8)	5 (2.7)	7 (3.6)		

Sex, *N* (%)					
Male	55 (32.7)	76 (40.4)	63 (32.5)	*χ* ^2^	0.190
Female	113 (67.3)	112 (59.6)	131 (67.5)		

Marital status, *N* (%)					
Single	68 (40.5)	86 (45.7)	80 (41.2)	*χ* ^2^	0.544
Married	100 (59.5)	102 (54.3)	114 (58.8)		

Educational level, *N* (%)					
Noneducated	2 (1.2)	2 (1.1)	6 (3.1)	*χ* ^2^	0.739
<12 years	59 (35.1)	61 (32.4)	66 (34.0)		
=12 years (diploma)	50 (29.8)	61 (32.4)	57 (29.4)		
Associate of science	12 (7.1)	13 (6.9)	14 (7.2)		
Bachelor of science	36 (21.4)	35 (18.6)	34 (17.5)		
Master of science	5 (3.0)	8 (4.3)	13 (6.7)		
Doctorate	4 (2.4)	8 (4.3)	4 (2.1)		

**Table 2:** Baseline characteristics and preoperative conditions in the three study arms.

Preoperative factors	RCT (*n* = 168)	PMTA (*n* = 188)	PCEM (*n* = 194)	Test	*P* value
Pain intensity, 0–9 scale (mean ± SE)	3.95 ± 0.21	3.95 ± 0.20	3.94 ± 0.19	ANOVA	0.998
*95% Confidence Interval (CI) for Mean*	3.54–4.37	3.56–4.35	3.55–4.33		

Distribution of teeth, *N* (%)					
Maxilla	76 (45.2)	87 (46.3)	88 (45.4)	*χ* ^2^	0.976
Mandible	92 (54.8)	101 (53.7)	106 (54.6)		

First molar	95 (56.5)	105 (55.9)	107 (55.2)	Fisher	0.806
Second molar	71 (42.3)	80 (42.6)	81 (41.8)		
Third molar	2 (1.2)	3 (1.6)	6 (3.1)		

Characteristics of teeth, *N* (%)					
With occlusal contact	155 (92.3)	168 (89.4)	175 (90.2)	*χ* ^2^	0.602
Without occlusal attrition	161 (95.8)	181 (96.3)	183 (94.3)	Fisher	0.881
Presence of coronal restoration	41 (24.4)	39 (20.7)	44 (22.7)	*χ* ^2^	0.714

Electric pulp testing (mean ± SE)	7.52 ± 0.70	6.64 ± 0.53	7.36 ± 0.63	ANOVA	0.570
*N* (%)	166 (98.8)	188 (100.0)	192 (99.0)	Fisher	0.468

Cold testing (normal response), *N* (%)	124 (73.8)	132 (70.2)	142 (73.2)	Fisher	0.388

Widening of PDL, *N* (%)	11 (6.5)	13 (6.9)	12 (6.2)	*χ* ^2^	0.969

Symptomatic irreversible pulpitis, *N* (%)	37 (22.0)	49 (26.6)	45 (23.2)	Fisher	0.388
Symptomatic apical periodontitis, *N* (%)	23 (13.7)	23 (12.2)	20 (10.4)	*χ* ^2^	0.662

**Table 3:** Comparison of intraoperative conditions in the three study arms.

Intraoperative factors	RCT (*n* = 168)	PMTA (*n* = 188)	PCEM (*n* = 194)	Test	*P* value
Bleeding nature, *N* (%)					
Not recorded	31 (18.5)	30 (16.0)	26 (13.4)		
Oozing	39 (23.2)	64 (12.0)	55 (28.4)	Kruskal	0.105
Normal	82 (48.8)	73 (38.8)	81 (41.8)	Wallis	
Profound	16 (9.5)	21 (11.2)	32 (16.4)		

Hemostasis: not achieved, *N* (%)					
Using chlorhexidine (5 min)	NA	83 (44.1)	91 (55.9)	*χ* ^2^	0.493
Using sodium hypochlorite (30 sec)	NA	3^*∗*^(1.6)	5^*∗*^(2.6)	Fisher	0.723

Treatment length, minutes (mean ± SE)					
Endodontic procedure	69.73 ± 2.60	35.37 ± 1.27	33.62 ± 1.19	ANOVA	<0.001
*95% Confidence Interval (CI) for Mean*	64.59–74.86	32.87–37.88	31.26–35.97		
Restorative procedure	39.00 ± 1.29	42.17 ± 1.80	41.59 ± 1.66	ANOVA	0.356
*95% Confidence Interval (CI) for Mean*	36.43–4157	38.61–45.74	38.30–44.87		

Two-visit treatment, *N* (%)	49 (29.1)	1 (0.05)	1 (0.05)	*χ* ^2^	<0.001

^*∗*^All the teeth have achieved hemostasis after 10 min application of NaOCl.

**Table 4:** Comparison of postoperative conditions in the three study arms.

Postoperative factors	RCT (*n* = 168)	PMTA (*n* = 188)	PCEM (*n* = 194)	Test	*P* value
Cold test, *N* (%)					
Negative response	NA	139 (73.9)	151 (77.8)	*χ* ^2^	0.373
Positive response	NA	49 (26.1)	43 (22.2)		

Electric pulp test, positive response, *N* (%)	NA	99 (52.7)	93 (47.9)	*χ* ^2^ * t*-test	0.356
Mean ± SE	NA	4.91 ± 0.62	5.09 ± 0.72		0.857
*95% Confidence Interval (CI) for Mean*	NA	3.70–6.13	3.65–6.53		

Positive percussion test, *N* (%)	10 (6.0)	11 (5.9)	15 (7.7)	*χ* ^2^	0.712

Analgesic intake					
Mean number of tablets/persons ± SD	1.51 ± 0.13	1.50 ± 0.12	1.61 ± 0.14	ANOVA	0.809
*95% Confidence Interval (CI) for Mean*	1.24–1.77	1.26–1.74	1.32–1.89		
Analgesics used, number of people (%)	108 (64.3)	117 (62.2)	128 (66.0)	*χ* ^2^	0.747

Drug, *N* (%)					
Acetaminophen (w/o codeine)	21 (19.4)	31 (26.5)	29 (22.7)	Fisher	0.066
Gelofen	42 (38.9)	55 (47.0)	59 (46.1)		
Ibuprofen	37 (34.3)	31 (26.5)	34 (26.6)		
Others	8 (7.4)	0 (0.0)	6 (4.7)		

**Table 5:** Frequency distribution of pain severities [*N* (%)] during 7 postoperative days in the three study arms.

Day	Arm	PI category				Reporting pain (%)	*P* value
		Pain-free	Mild	Moderate	Severe		
Baseline	RCT^*∗*^	27 (16.1)	46 (27.4)	58 (34.5)	37 (22.0)	83.9	0.998
	PMTA	30 (16.0)	53 (28.2)	67 (35.6)	38 (20.2)	84.0	
	PCEM^†^	28 (14.4)	56 (28.9)	68 (35.1)	42 (21.6)	85.6	

6 h	RCT	32 (19.0)	77 (45.8)	46 (27.4)	13 (7.7)	81.0	0.337
	PMTA	33 (17.6)	73 (38.8)	68 (36.2)	14 (7.4)	82.4	
	PCEM	43 (22.2)	72 (37.1)	58 (29.9)	21 (10.8)	77.8	

12 h	RCT	60 (35.7)	61 (36.3)	38 (22.6)	9 (5.4)	64.3	0.585
	PMTA	67 (35.6)	78 (41.5)	39 (20.7)	4 (2.1)	64.4	
	PCEM	66 (34.0)	80 (41.2)	37 (19.1)	11 (5.7)	66.0	

18 h	RCT	86 (51.2)	59 (35.1)	18 (10.7)	5 (3.0)	48.8	0.593
	PMTA	92 (48.9)	73 (38.8)	20 (10.6)	3 (1.6)	51.1	
	PCEM	91 (46.9)	70 (36.1)	23 (11.9)	10 (5.2)	53.1	

24 h	RCT	100 (59.5)	46 (27.4)	18 (10.7)	4 (2.4)	40.5	0.918
	PMTA	115 (61.2)	53 (28.2)	17 (9.0)	3 (1.6)	38.8	
	PCEM	109 (56.2)	60 (30.9)	19 (9.8)	6 (3.1)	43.8	

36 h	RCT	111 (66.1)	38 (22.6)	16 (9.5)	3 (1.8)	33.9	0.056
	PMTA	131 (69.7)	41 (21.8)	15 (8.0)	1 (0.5)	30.3	
	PCEM	126 (64.9)	56 (28.9)	6 (3.1)	6 (3.1)	35.1	

3 d	RCT	125 (74.4)	31 (18.5)	12 (7.1)	0( 0.0)	25.6	0.148
	PMTA	143 (76.1)	35 (18.6)	7 (3.7)	3 (1.6)	23.9	
	PCEM	148 (76.3)	41 (21.1)	4 (2.1)	1 (0.5)	23.7	

4 d	RCT	132 (78.6)	31 (18.5)	5 (3.0)	0 (0.0)	21.4	0.508
	PMTA	155 (82.4)	24 (12.8)	8 (4.3)	1 (0.5)	17.6	
	PCEM	158 (81.4)	30 (15.5)	4 (2.1)	2 (1.0)	18.6	

5 d	RCT	137 (81.5)	25 (14.9)	5 (3.0)	1 (0.6)	18.5	0.993
	PMTA	155 (82.4)	26 (13.8)	6 (3.2)	1 (0.5)	17.6	
	PCEM	164 (84.5)	23 (11.9)	6 (3.1)	1 (0.5)	15.5	

6 d	RCT	143 (81.5)	23 (13.7)	1 (0.6)	1 (0.6)	18.5	0.719
	PMTA	162 (86.2)	19 (10.1)	5 (2.7)	2 (1.1)	13.8	
	PCEM	163 (84.0)	24 (12.4)	5 (2.6)	2 (1.0)	16.0	

7 d	RCT	147 (87.5)	19 (11.3)	1 (0.6)	1 (0.6)	12.5	0.834
	PMTA	166 (88.3)	18 (9.6)	4 (2.1)	0 (0.0)	11.7	
	PCEM	169 (87.1)	21 (10.8)	3 (1.5)	1 (0.5)	12.9	

**Table 6:** Mean and median time (hrs) to pain-free status in the study arms.

Groups	Mean ± SE (hrs)	95% CI for mean	Median ± SE (hrs)	95% CI for median	Test	*P* value
RCT	54.54 ± 4.58	45.56–63.51	18.0 ± 1.89	14.28–21.72	Log rank	0.746
PMTA	55.57 ± 4.26	47.22–63.93	24.0 ± 1.94	20.19–27.81		
PCEM	58.71 ± 4.23	50.42–67.00	24.0 ± 3.29	17.54–30.46		

## Data Availability

The data used to support the findings of this study are available from the corresponding author upon reasonable request.
